# Effects of Subchronic Administrations of Vortioxetine, Lurasidone, and Escitalopram on Thalamocortical Glutamatergic Transmission Associated with Serotonin 5-HT7 Receptor

**DOI:** 10.3390/ijms22031351

**Published:** 2021-01-29

**Authors:** Motohiro Okada, Ryusuke Matsumoto, Yoshimasa Yamamoto, Kouji Fukuyama

**Affiliations:** Department of Neuropsychiatry, Division of Neuroscience, Graduate School of Medicine, Mie University, Tsu 514-8507, Japan; matsumoto-r@clin.medic.mie-u.ac.jp (R.M.); Yshimasa.home6041@outlook.jp (Y.Y.); k-fukuyama@clin.medic.mie-u.ac.jp (K.F.)

**Keywords:** lurasidone, serotonin, vortioxetine, microdialysis, serotonin

## Abstract

The functional suppression of serotonin (5-HT) type 7 receptor (5-HT7R) is forming a basis for scientific discussion in psychopharmacology due to its rapid-acting antidepressant-like action. A novel mood-stabilizing atypical antipsychotic agent, lurasidone, exhibits a unique receptor-binding profile, including a high affinity for 5-HT7R antagonism. A member of a novel class of antidepressants, vortioxetine, which is a serotonin partial agonist reuptake inhibitor (SPARI), also exhibits a higher affinity for serotonin transporter, serotonin receptors type 1A (5-HT1AR) and type 3 (5-HT3R), and 5-HT7R. However, the effects of chronic administration of lurasidone, vortioxetine, and the selective serotonin reuptake inhibitor (SSRI), escitalopram, on 5-HT7R function remained to be clarified. Thus, to explore the mechanisms underlying the clinical effects of vortioxetine, escitalopram, and lurasidone, the present study determined the effects of these agents on thalamocortical glutamatergic transmission, which contributes to emotional/mood perception, using multiprobe microdialysis and 5-HT7R expression using capillary immunoblotting. Acute local administration of a 5-HT7R agonist and antagonist into the mediodorsal thalamic nucleus (MDTN) enhanced and reduced thalamocortical glutamatergic transmission, induced by N-methyl-D-aspartate (NMDA)/glutamate receptor inhibition in the reticular thalamic nucleus (RTN). Acute local administration of a relevant therapeutic concentration of vortioxetine and lurasidone into the MDTN suppressed the thalamocortical glutamatergic transmission via 5-HT7R inhibition, whereas that of escitalopram activated 5-HT7R. Subchronic administration of effective doses of vortioxetine and lurasidone (for 7 days) reduced the thalamocortical glutamatergic transmission, but escitalopram did not affect it, whereas subchronic administration of these three agents attenuated the stimulatory effects of the 5-HT7R agonist on thalamocortical glutamatergic transmission. Subchronic administration of effective doses of vortioxetine, lurasidone, and escitalopram downregulated the 5-HT7R expression of the plasma membrane in the MDTN; the 5-HT7R downregulation induced by vortioxetine and lurasidone was observed at 3 days, but that induced by escitalopram required a longer duration of 7 days. These results indicate that chronic administration of vortioxetine, escitalopram, and lurasidone generate downregulation of 5-HT7R in the thalamus; however, the direct inhibition of 5-HT7R associated with vortioxetine and lurasidone generates more rapid downregulation than the indirect elevation of the extracellular serotonin level via serotonin transporter inhibition by escitalopram.

## 1. Introduction

In accordance with the serotonin hypothesis regarding the pathophysiology of mood disorders postulated by Coppen in 1967 [1], modern psychopharmacology has endeavoured to overcome mood disorders via the development of various types of antidepressants, including tricyclic antidepressants, monoamine oxidase inhibitors, selective serotonin re-uptake inhibitors (SSRIs), norepinephrine/serotonin re-uptake inhibitors, noradrenergic and specific serotonergic antidepressants, and serotonin partial agonist reuptake inhibitors (SPARIs). Accumulating clinical and preclinical findings provide us with a number of candidate pathophysiologies of SSRIs, associated with dysfunctions of developmental plasticity, brain-derived neurotrophic factors, and tripartite synaptic transmission [2,3]; however, it has been well established that the inhibition of the serotonin transporter contributes to the primary mechanisms behind the antidepressive action of SSRIs as a first-line antidepressant [4]. Acute administration of a therapeutic-relevant dose of an SSRI increases the extracellular serotonin levels in the serotonergic terminal regions, such as the medial prefrontal cortex (mPFC) and hippocampus [5,6,7]; however, the increased extracellular serotonin level generates an acutely somatodendritic type 1A serotonin receptor (5-HT1AR)-mediated reduction in monoaminergic transmission [6,8,9,10]. Contrary to the acute phase, chronic administration of an SSRI desensitises or downregulates inhibitory 5-HT1AR, resulting in enhanced serotonergic transmission in the chronic phase [11]. It has been well established that the downregulation of 5-HT1AR induced by chronic SSRI administration contributes to the mechanisms behind the antidepressive action of SSRIs; however, complete long-term adaptation of 5-HT1AR requires several weeks [12]. In particular, an SSRI usually requires several weeks to have beneficial antidepressant effects [13]. Based on these clinical and preclinical findings, downregulation or desensitisation of 5-HT1AR, which is followed by an enhanced serotonergic tone induced by the SSRI, has been proposed to explain the clinical delay in the antidepressive effect of SSRIs [11].

A non-competitive N-methyl-D-aspartate/glutamate receptor (NMDAR) antagonist, esketamine (a racemic enantiomer of ketamine), was approved by the Food and Drug Administration and the European Medicines Agency in 2019 for the treatment of antidepressant-resistant depressive disorders [14]. Importantly, esketamine/ketamine holds the potential to improve monoaminergic antidepressant-resistant dysfunction of emotional perception/cognition in mood disorders with rapid action [14]. Similarly, initial behavioural studies reported that the serotonin type 7 receptor (5-HT7R) antagonists (R)-3-((2-(2-(4-Methylpiperidin-1-yl)ethyl)pyrrolidin-1-yl)sulfonyl)phenol (SB269970) and 1-benzyl-3-(4-chlorophenyl)-5,6,7,8-tetrahydro-4H-pyrazolo (3,4-d) azepine (JNJ18038683) produced antidepressant-like effects and promoted the antidepressant effects of several conventional antidepressants (citalopram, imipramine, desipramine, and moclobemide) [15,16,17]. The rapid-acting antidepressant-like effects of the 5-HT7R antagonists [18] led many psychiatrists to expect the development of a novel rapid-onset monoaminergic antidepressant class superior to conventional antidepressants in clinics. Unfortunately, the durations required for the onset of the antidepressant effects of lurasidone and vortioxetine seem to be equivalent to those for conventional antidepressants [19,20,21,22,23]; however, a recent clinical trial demonstrated that both intravenous and oral administrations of vortioxetine resulted in significant improvements in depression and anxiety after three days [23]. Therefore, the rapid-acting antidepressant and anxiolytics actions of 5-HT7R antagonism have not been completely ruled out and are worth reassessing after future clinical findings have been accumulated.

Aside from the clinical advantages of SSRIs, the increased extracellular serotonin level induced by an SSRI also provides the initial adverse effects of the SSRI, such as jitteriness/anxiety syndrome (including somatic anxiety, heightened anxiety, anger, and agitation) [24,25]. An initial systematic review indicated that the incidence rates vary widely, from 4 to 65%, among individuals commencing SSRI medication [24]. A recent meta-analysis study reported that the mean rating of somatic anxiety was significantly higher in the SSRI group than the placebo group at one week after medication commenced (9.3% vs. 6.7%) [25]. Contrary to SSRIs, the incidence of aggravation of anger/agitation associated with vortioxetine, as determined using the Food and Drug Administration Adverse Event Reporting System database, was 0.002–3.5% in the vortioxetine-induced adverse reaction pool [26]. Although the details of the antidepressant-induced jitteriness/anxiety syndrome (its incidence and mechanism) have not yet been clarified, the current clinical findings suggest that vortioxetine does not pose a higher risk of jitteriness/anxiety syndrome compared to conventional SSRIs [26]. The binding profiles of vortioxetine [27] suggest that the impact of the antagonism of serotonin type 3 receptor (5-HT3R) and 5-HT7R by vortioxetine probably contributes to its lower risk of jitteriness/anxiety syndrome.

A number of preclinical studies reported that activation of presynaptic 5-HT1AR and inhibition of presynaptic 5-HT3R have an important antidepressant-like action [28]. Aside from this antidepressant-like action, inhibition of 5-HT3R contributes to anxiolytic-like action [28,29,30,31], whereas both inhibition and activation of 5-HT1AR result in the exhibition of anxiolytic-like action [28]. Regarding neuronal adaptation of 5-HT3R induced by antidepressants, chronic administration of a therapeutic-relevant dose of both escitalopram and vortioxetine suppressed 5-HT3R function; however, while escitalopram desensitised/downregulated 5-HT3R function, vortioxetine did not affect it [6,32]. Taken together with preclinical findings regarding 5-HT3R, these results indicate that both escitalopram and vortioxetine chronically inhibit the serotonergic transmission associated with 5-HT3R; however, the mechanisms of 5-HT3R suppression induced by escitalopram and vortioxetine are not identical. Escitalopram chronically suppresses 5-HT3R via activation of 5-HT1AR and 5-HT3R induced by elevation of the extracellular serotonin level, whereas vortioxetine acutely and chronically inhibits 5-HT3R via its direct 5-HT3R antagonism [6]. This difference between the chronic effects of escitalopram and vortioxetine on 5-HT3R functional suppression probably contributes to the incidences of jitteriness/anxiety syndrome induced by SSRIs and vortioxetine.

On the other hand, the impacts of psychopharmacological 5-HT7R antagonism on several types of psychiatric disorders should be reassessed. Accumulating preclinical demonstrations have reported that inhibition of postsynaptic 5-HT7R has important antidepressant-like action [28]; however, both inhibition and activation of 5-HT7R result in the exhibition of anxiolytic-like action [28]. Indeed, in contrast to initial behavioural studies, recent behavioural studies involving acute administration of the selective 5-HT7R antagonists 2a-(4-(4-phenyl-3,6-dihydro-2H-pyridin-1-yl)butyl)-1,3,4,5-tetrahydrobenzo(cd)indol-2-one (DR4004) and SB269970 indicated antipsychotic-like properties in a phencyclidine-induced hyperactivity assay and anxiolytic/anti-stress effects in a stress-induced cGMP assay, but neither significant anxiolytic-like (elevated plus maze and Vogel conflict tests) nor antidepressant-like (tail-suspension test) efficacy was observed [33]. Additionally, another behavioural study also demonstrated that 5-HT7R knock-out mice displayed significantly different responses in fear conditioning, shock-probe burying, novelty-suppressed feeding, punishment memory, forced swim test, and amphetamine hyperactivity assays compared to wild-type mice; however, there was no consistency in the direction of behavioural effects across genotypes or across assays [34].

A novel mood-stabilising atypical antipsychotic drug, lurasidone, and a SPARI, vortioxetine, block 5-HT7R [6,35,36,37]. However, the chronic effects of vortioxetine and lurasidone on 5-HT7R function remain to be clarified. The pharmacological profile of 5-HT7R exhibits atypical features in the serotonin receptor family. In particular, subacute exposure to both 5-HT7R agonists and antagonists, such as SB269970, clozapine, and olanzapine, downregulates 5-HT7R expression in the plasma membrane [38]. Additionally, the binding profiles of vortioxetine using recombinant cell lines expressing human and rat serotonin receptor subtypes are not identical [39,40]. Vortioxetine is a high-affinity inhibitor of human 5-HT3R (Ki = 3.7 nM), 5-HT7R (Ki = 19 nM), and serotonin transporter (Ki = 1.6 nM), and an agonist of 5-HT1AR (Ki = 15 nM); it also has a high affinity to rat 5-HT3R (Ki = 1.1 nM) and serotonin transporter (Ki = 8.6 nM), but a lower affinity to 5-HT1AR (Ki = 230 nM) and 5-HT7R (Ki = 200 nM) [39,40]. Considering the lower affinity of vortioxetine to rat 5-HT1AR and 5-HT7R compared to humans, it has been speculated that the chronic effects of vortioxetine on 5-HT1AR and 5-HT7R have not been adequately targeted in pharmacodynamic studies.

Several clinical studies reported that the 5-HT7R variants are not associated with response to atypical antipsychotics in schizophrenia [41,42], and a significant association was found between responses to positive and negative symptoms with lurasidone and functional polymorphism of 5-HT1AR, but not that of 5-HT7R [43]. Aside from its antipsychotic effects, a functional promoter single-nucleotide polymorphism in the 5-HTR7 gene, rs7905446, which leads to greater 5-HT7R expression [44], was associated with better response to SSRIs in individuals with bipolar disorder and major depression [44]. Therefore, these clinical findings suggest that 5-HT7R antagonism possibly plays an important role in antidepressant-like action.

5-HT7R is highly expressed in regions relevant to cognitive function, such as the thalamus, as well as in the hypothalamus, hippocampus, prefrontal cortex, striatal complex, amygdala, and dorsal raphe nucleus [45,46,47,48]. Over the last two decades, preclinical studies have accumulated various findings, highlighting 5-HT7R as a key player in the regulation of mood, memory processing, cognition, and emotional perception, as demonstrated by various experiments using selective 5-HT7R antagonists and 5-HT7R knock-out mice models [28,49]. Moreover, the predominant expression of 5-HT7R in the limbic regions supports the hypothesis that 5-HT7R contributes to the regulation of memory processing, cognition, and emotional perception in association with several types of cognitive domains [35,50,51].

Based on the previous clinical and preclinical findings, to clarify the mechanisms of clinical action of the acute and chronic effects of vortioxetine (a SPARI) and lurasidone (a mood-stabilising atypical antipsychotic) associated with 5-HT7R, the present study determined the effects of subchronic administrations of these two agents on transmission in comparison with one of the most selective serotonin transporter inhibitors, escitalopram [6,52]. The first part of the study determined the acute effects of vortioxetine, escitalopram, and lurasidone on thalamocortical glutamatergic transmission associated with 5-HT1AR and 5-HT7R regulation. The second part of the study determined the subchronic effects of vortioxetine, escitalopram, and lurasidone on thalamocortical glutamatergic transmission associated with 5-HT7R regulation. The third part of the study determined the effects of subacute and subchronic administration of vortioxetine, escitalopram, and lurasidone on thalamic 5-HT7R expression.

## 2. Results

### 2.1. Effects of Perfusion with a 5-HT7R Agonist, Antagonist, Vortioxetine, Escitalopram, and Lurasidone into the MDTN on MK801-Induced L-Glutamate Release in the mPFC (Study 1)

**Figure 1:**
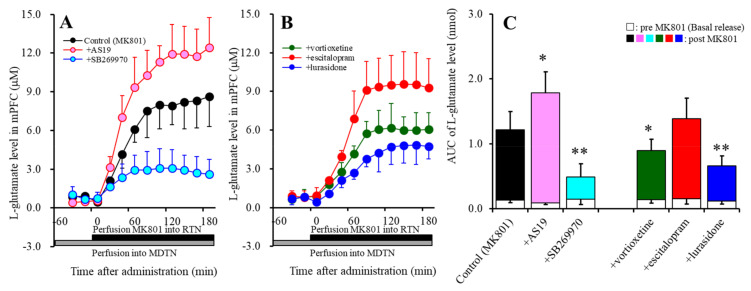
Effects of local administrations of a 5-HT7R agonist, (2S)-(+)-5-(1,3,5-trimethylpyrazol-4-YL)-2-(dimethylamino)tetralin (AS19: 5 μM), an antagonist, (R)-3-((2-(2-(4-Methylpiperidin-1-yl)ethyl)pyrrolidin-1-yl)sulfonyl)phenol (SB269970: 10 μM), 50 μM vortioxetine, 10 μM escitalopram, and 1 μM lurasidone into the mediodorsal thalamic nucleus (MDTN) on L-glutamate release in the medial prefrontal cortex (mPFC) induced by perfusion with dizocilpine (MK801: 10 μM) into the reticular thalamic nucleus (RTN). Ordinates (Panels (**A**) and (**B**)): mean ± SD (*n* = 6) of the extracellular levels of L-glutamate (μM) in the mPFC. Abscissa: time after administration of MK801 (min). Closed and grey columns indicate perfusion with MK-801 into the RTN and 5-HT7 receptor agents (AS19, SB269970, vortioxetine, escitalopram, and lurasidone) into the MDTN. Panel (**C**) indicates the area under curve (AUC) value of the extracellular levels of L-glutamate in the mPFC (nmol) after perfusion with 10 μM MK801 from 20 to 180 min of Panels A and B. Especially, the opened columns indicate the AUC values prior to the MK801-evoked stimulation (basal release). * *p* < 0.05, ** *p* < 0.01: significantly different from the control (perfusion with 10 μM MK801 into the RTN alone) by multivariate analysis of variance (MANOVA) with Tukey’s post-hoc test.

Previous studies demonstrated that perfusion with MK801 into the RTN increased L-glutamate release in the mPFC due to the GABAergic disinhibition in the intra-thalamic pathway (from RTN to MDTN) [6,35,53,54,55,56]. The threshold concentration of perfusion with MK801 into the RTN for an increase in L-glutamate release in the mPFC was lower than 1 μM [54]. In the present study, perfusion with 10 μM MK801 into the RTN (MK801-evoked stimulation) also increased L-glutamate release in the mPFC (MK801-evoked L-glutamate release) (Figure 1A,C).

To study the effects of 5-HT7R on thalamocortical glutamatergic transmission, prior to MK801-evoked stimulation, the MDTN, which is one of the most predominant expression regions of 5-HT7R [6,35,46], was locally administered with a 5-HT7R agonist, (2S)-(+)-5-(1,3,5-trimethylpyrazol-4-YL)-2-(dimethylamino)tetralin (AS19: 5 μM) [6,35,57], or antagonist, SB269970 (10 μM) [6,35,57]. Activation of 5-HT7R in the MDTN by perfusion with 5 μM AS19 enhanced the MK801-evoked L-glutamate release (F_AS19_ (1,10) = 10.0 (*p* < 0.05); F_Time_ (2.7,26.8) = 196.9 (*p* < 0.01); and F_AS19*Time_ (2.7,26.8) = 7.5 (*p* < 0.01)), *p* < 0.05; whereas, inhibition of 5-HT7R in the MDTN by perfusion with 10 μM SB269970 suppressed the MK801-evoked L-glutamate release (F_SB269970_ (1,10) = 24.7 (*p* < 0.01); F_Time_ (2.3,22.9) = 82.3 (*p* < 0.01); and F_SB269970*Time_ (2.3,22.9) = 32.1 (*p* < 0.01)), *p* < 0.01 (Figure 1A,C).

Previous studies, using brain slices, demonstrated that direct application of 10 μM escitalopram and 20 μM vortioxetine affected neuronal firing [58,59]. Perfusion with 1 μM lurasidone into the MDTN inhibited the MK-801-evoked L-glutamate release [35]. Based on these previous observations, in the present study, 20 μM vortioxetine, 10 μM escitalopram, and 1 μM lurasidone were perfused into the MDTN. Both perfusion with 20 μM vortioxetine (F_Vortioxetine_ (1,10) = 5.5 (*p* < 0.05); F_Time_ (2.7,26.7) = 122.7 (*p* < 0.01); F_Vortioxetine*Time_ (2.7,26.7) = 3.7 (*p* < 0.05)), *p* < 0.05, and 1 μM lurasidone (F_Lurasidone_ (1,10) = 17.4 (*p* < 0.01); F_Time_ (2.4,24.1) = 111.1 (*p* < 0.01); and F_Lurasidone*Time_ (2.4,24.1) = 9.7 (*p* < 0.01)), *p* < 0.01, into the MDTN inhibited the MK801-evoked L-glutamate release, whereas perfusion with 10 μM escitalopram did not affect the MK801-evoked L-glutamate release (Figure 1B,C).

**Figure 2:**
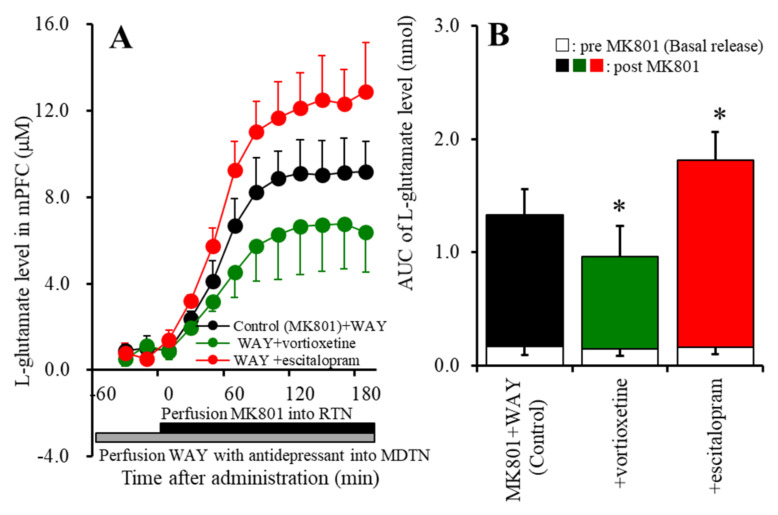
Under the blockade of serotonin type 1A receptor (5-HT1AR) in the MDTN by perfusion with 10 μM WAY100635, and the effects of the local administration of 20 μM vortioxetine and 10 μM escitalopram into the MDTN on MK801-evoked L-glutamate release in the mPFC. Ordinates (Panel (**A**)): mean ± SD (*n* = 6) of the extracellular levels of L-glutamate (μM) in the mPFC. Abscissa: time after administration of MK801 (min). Closed and grey columns indicate perfusion with 10 μM MK801 into the RTN and 10 μM WAY100635 with antidepressants (20 μM vortioxetine or 10 μM escitalopram) into the MDTN. Panel (**B**) indicates the AUC value of the extracellular levels of L-glutamate in the mPFC (nmol) after perfusion with 10 μM MK801 from 20 to 180 min of Panel (**A**). Especially, the opened columns indicate the AUC values prior to the MK801-evoked stimulation (basal release). * *p* < 0.05: significantly different from the control (perfusion of 10 μM WAY100635 into the MDTN with 10 μM MK801 into the RTN) by MANOVA with Tukey’s post-hoc test.

To clarify the effects of 5-HT1AR activated by the elevation of the extracellular serotonin level induced by escitalopram and vortioxetine, perfusion with a 5-HT1AR antagonist, WAY100635 (10 μM) [8,60], into the MDTN happened prior to the MK801-evoked stimulation. Perfusion with 10 μM WAY100635 did not affect the MK801-evoked L-glutamate release (Figure 1A,C or Figure 2A,B). Under the blockade of 5-HT1AR in the MDTN, perfusion with 20 μM vortioxetine into the MDTN remained to decrease the MK81-evoked L-glutamate release (F_Vortioxetine_ (1,10) = 6.4 (*p* < 0.05); F_Time_ (1.8,18.1) = 153.3 (*p* < 0.01); and F_Vortioxetine*Time_ (1.8,18.1) = 5.2 (*p* < 0.05)), *p* < 0.05; whereas, perfusion with 10 μM escitalopram into the MDTN conversely increased the MK801-evoked L-glutamate release (F_Escitalopram_ (1,10) = 11.8 (*p* < 0.01); F_Time_ (3.0,29.5) = 323.9 (*p* < 0.01); and F_Escitalopram*Time_ (3.0,29.5) = 8.5 (*p* < 0.01)), *p* < 0.05 (Figure 2A,C).

### 2.2. Effects of the Subchronic, Systemic Administration of Effective Doses of Vortioxetine, Escitalopram, and Lurasidone on MK801-Induced L-Glutamate Release in the mPFC (Study 2)

#### 2.2.1. Effects of the Subchronic Administration of Vortioxetine, Escitalopram, and Lurasidone on MK801-Induced L-Glutamate Release in the mPFC

The minimum effective doses of systemic administrations of vortioxetine and escitalopram on the extracellular serotonin level are 2.5 mg/kg/day and 5 mg/kg/day, respectively [6,35]. Additionally, the effective dose of subchronic administration of lurasidone is 3 mg/kg/day [6]. According to these previous reports, in the present study, to study the subchronic effects of vortioxetine, escitalopram, and lurasidone, each rat was administered vortioxetine (2.5 mg/kg/day), escitalopram (5 mg/kg), or lurasidone (3 mg/kg/day) for 7 days using a subcutaneous osmotic pump (2ML_1, Alzet, Cupertino, CA, USA). Pharmacokinetic studies reported that the half-life of vortioxetine, escitalopram, and lurasidone were 8, 2, and 9 h [61,62,63]. Based on these pharmacodynamic and pharmacokinetic data, after 24 h of stopping the subchronic administration of escitalopram, vortioxetine, and lurasidone, the perfusion experiment in the mPFC and RTN was started.

**Figure 3:**
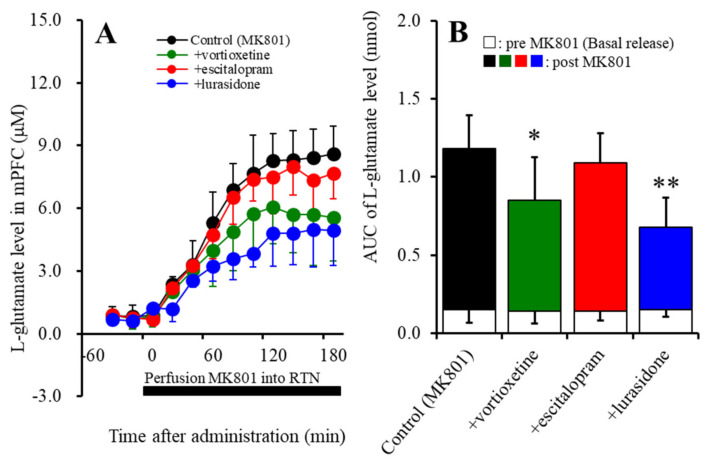
Effects of subchronic, systemic administration of effective doses of vortioxetine (2.5 mg/kg/day), escitalopram (5 mg/kg/day), and lurasidone (3 mg/kg/day) for 7 days on 10 μM MK801-evoked L-glutamate release in the mPFC. Ordinates (Panel (**A**)): mean ± SD (*n* = 6) of the extracellular levels of L-glutamate (μM) in the mPFC. Abscissa: time after administration of MK801 (min). Closed column indicates perfusion with 10 μM MK801 into the RTN. Panel (**B**) indicates the AUC value of the extracellular levels of L-glutamate in the mPFC (nmol) after perfusion with 10 μM MK801 from 20 to 180 min of Panel (A). Especially, the opened columns indicate the AUC values prior to the MK801-evoked stimulation (basal release). * *p* < 0.05, ** *p* < 0.01: significantly different from the control (perfusion with 10 μM MK801 into the RTN) by MANOVA with Tukey’s post-hoc test.

Subchronic administration of the therapeutic-relevant doses of vortioxetine, escitalopram, and lurasidone did not affect basal L-glutamate release in the mPFC (Figure 2A,B). Both subchronic administrations of vortioxetine (2.5 mg/kg/day for 7 days) (F_Vortioxetine_ (1,10) = 5.2 (*p* < 0.05); F_Time_ (3.4,34.3) = 99.8 (*p* < 0.01); and F_Vortioxetine*Time_ (3.4,34.3) = 5.5 (*p* < 0.01)), *p* < 0.05, and lurasidone (3 mg/kg/day for 7 days) (F_Lurasidone_ (1,10) = 17.7 (*p* < 0.01); F_Time_ (4.2,41.7) = 138.6 (*p* < 0.01); and F_Lurasidone*Time_ (4.2,41.7) = 14.2 (*p* < 0.01)), *p* < 0.01, reduced the MK801-evoked L-glutamate release, whereas escitalopram (5 mg/kg/day for 7 days) did not affect it (Figure 3A,B).

#### 2.2.2. Effects of Subchronic, Systemic Administration of Effective Doses of Vortioxetine, Escitalopram, and Lurasidone on 5-HT7R Associated with MK801-Induced L-Glutamate Release in the mPFC

To study the inhibitory effects of the subchronic administration of therapeutic-relevant doses of vortioxetine (2.5 mg/kg/day), escitalopram (5 mg/kg), or lurasidone (3 mg/kg/day) for 7 days, using an osmotic pump, on MK801-evoked L-glutamate release associated with 5-HT7R, 24 h after stopping the subchronic administrations, the perfusion experiments in the mPFC, MDTN, and RTN was started. After the confirming the stabilisation of the extracellular L-glutamate levels in the mPFC, the perfusion medium in the MDTN commenced with the MRS containing 5 μM AS19. After confirming the stabilisation of the extracellular L-glutamate levels in the mPFC, the perfusion medium in the RTN commenced with the MRS containing 10 μM MK801.

**Figure 4:**
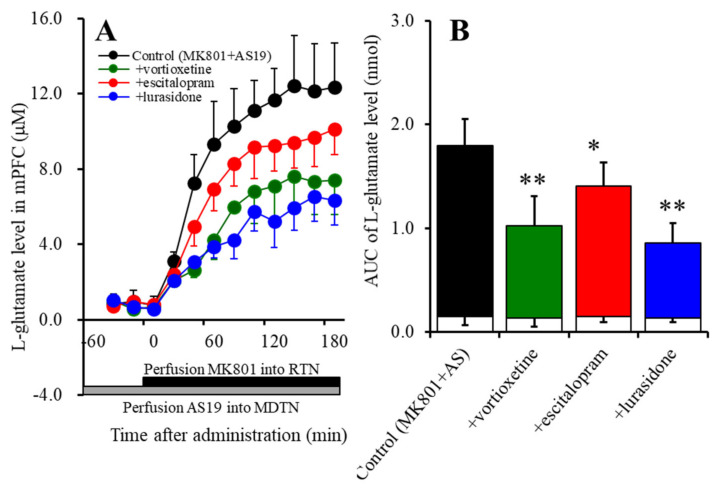
Effects of the subchronic, systemic administration of effective doses of vortioxetine (2.5 mg/kg/day), escitalopram (5 mg/kg/day), and lurasidone (3 mg/kg/day) for 7 days on enhanced 10 μM MK801-evoked L-glutamate release in the mPFC induced by 5 μM AS19. Ordinates (Panel (**A**)): mean ± SD (*n* = 6) of the extracellular levels of L-glutamate (μM) in the mPFC. Abscissa: time after administration of MK801 (min). Closed and grey columns indicate perfusion with 10 μM MK801 into the RTN and 5 μM AS19 into the MDTN, respectively. Panel (**B**) indicates the AUC value of the extracellular levels of L-glutamate in the mPFC (nmol) after perfusion with 10 μM MK801 from 20 to 180 min of Panel (A). Especially, the opened columns indicate the AUC values prior to the MK801-evoked stimulation (basal release). * *p* < 0.05, ** *p* < 0.01: significantly different from the control (perfusion with 10 μM MK801 into the RTN) by MANOVA with Tukey’s post-hoc test.

Subchronic administration of therapeutic-relevant doses of vortioxetine, escitalopram, and lurasidone did not affect basal L-glutamate release in the mPFC (Figure 4A,B). Perfusion with 5 μM AS19 also did not affect basal L-glutamate release in the mPFC (Figure 4A,B). Both subchronic administration of vortioxetine (2.5 mg/kg/day for 7 days) (F_Vortioxetine_ (1,10) = 23.6 (*p* < 0.01); F_Time_(3.5,34.7) = 154.4 (*p* < 0.01); and F_Vortioxetine*Time_ (3.5,34.7) = 11.1 (*p* < 0.01)), *p* < 0.01, escitalopram (F_Escitalopram_ (1,10) = 5.8 (*p* < 0.05); F_Time_ (3.3,33.0) = 254.7 (*p* < 0.01); and F_Escitalopram*Time_ (3.3,33.0) = 3.8 (*p* < 0.01)), *p* < 0.05, and lurasidone (3 mg/kg/day for 7 days) (F_Lurasidone_ (1,10) = 38.3 (*p* < 0.01); F_Time_ (3.3,32.6) = 170.4 (*p* < 0.01); and F_Lurasidone*Time_ (3.3,32.6) = 24.8 (*p* < 0.01)), *p* < 0.01, suppressed the enhanced MK801-evoked L-glutamate release induced by the 5-HT7R agonist, 5 μM AS19 (Figure 4A,B).

### 2.3. Time-Dependent Effects of Subchronic Administration of Effective Doses of Vortioxetine, Escitalopram, and Lurasidone on Expression of 5-HT7R in the Thalamic Plasma Membrane Fraction (Study 3)

5-HT7R was downregulated by exposure to SB269970, clozapine, and olanzapine (5-HT7R inhibitor), for 24 h, in the 5-HT7R transfected human embryonic kidney 293 (HEK293) cells [38,64]. Therefore, from the results in Study 2, the subchronic administration of vortioxetine, escitalopram, and lurasidone attenuated the stimulatory effects of AS19 on MK801-evoked L-glutamate release, suggesting the possibility that subchronic administration of vortioxetine, escitalopram, and lurasidone leads to downregulation of 5-HT7R in the MDTN. The plasma membrane fractions of the rat thalamus [65] after subchronic administration of effective doses of vortioxetine (2.5 mg/kg/day), escitalopram (5 mg/kg/day), or lurasidone (3 mg/kg/day), using an osmotic pump for 3 or 7 days, were extracted by the Minute Plasma Membrane Protein Isolation Kit (Invent Biotechnologies, Plymouth, MN, USA).

**Figure 5:**
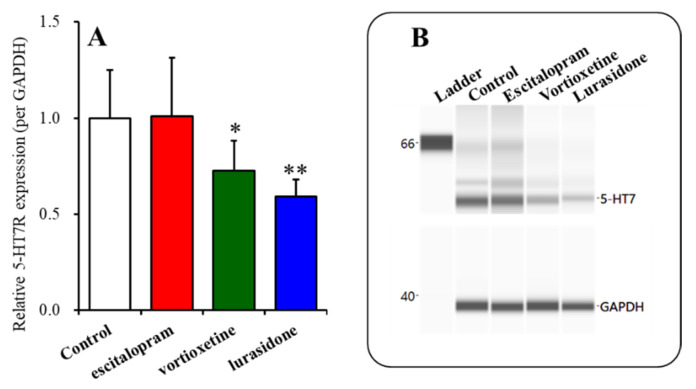
Effects of the subacute administration of effective doses of vortioxetine (2.5 mg/kg/day), escitalopram (5 mg/kg/day), and lurasidone (3 mg/kg/day) for 3 days on the expression of 5-HT7R in the thalamic plasma membrane fraction (Panel (**A**)). Ordinate: mean ± SD (*n* = 6) of the relative protein level of 5-HT7R in the thalamic plasma membrane fraction. Panel (**B**) indicates the pseudo-gel images using capillary immunoblotting. * *p* < 0.05, ** *p* < 0.01 vs. the control by Student’s *t*-test.

Subacute administrations of vortioxetine (2.5 mg/kg/day) (*p* < 0.05) and lurasidone (3 mg/kg/day) (*p* < 0.01) for 3 days decreased the 5-HT7R expression in the thalamic plasma membrane fractions, whereas subacute administrations of escitalopram (5 mg/kg) for 3 days did not affect it (Figure 5A,B).

**Figure 6:**
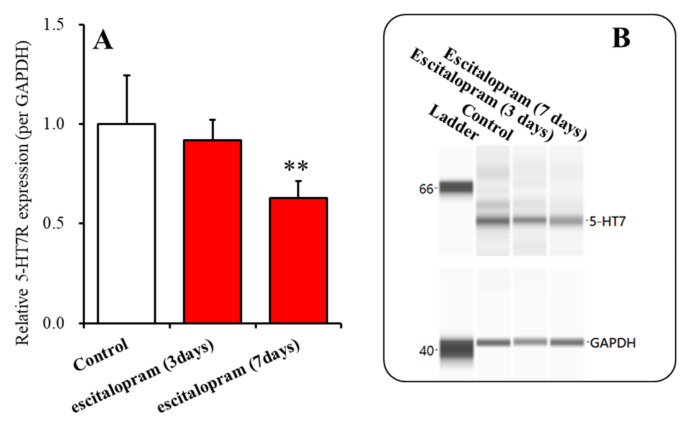
Time-dependent effects of subacute administration of effective dose of escitalopram (5 mg/kg/day) for 3 and 7 days on expression of 5-HT7R in the thalamic plasma membrane fraction (Panel (**A**)). Ordinate: mean ± SD (*n* = 6) of the relative protein level of 5-HT7R in the thalamic plasma membrane fraction. Panel (**B**) indicates the pseudo-gel images using capillary immunoblotting. ** *p* < 0.01 vs. control by one-way analysis of variance with Tukey’s post-hoc test.

Subacute administrations of escitalopram (5 mg/kg/day) for 3 days did not affect the 5-HT7R expression in the thalamic plasma membrane fractions, but subchronic administrations of escitalopram (5 mg/kg/day) for 7 days decreased it (Figure 6A,B).

## 3. Discussion

### 3.1. Effects of Acute Local Administration of Vortioxetine, Escitalopram and Lurasidone on Thalamocortical Glutamatergic Transmission

Activation of 5-HT1AR in the MDTN acutely suppressed thalamocortical glutamatergic transmission [6,14,35]; however, inhibition of 5-HT1AR enhanced thalamocortical glutamatergic transmission without affecting basal L-glutamate release in the mPFC (Figure 2). Therefore, the inhibitory effects of 5-HT1AR in the MDTN on the thalamocortical glutamatergic pathway are possibly due to phasic inhibition. Contrary to that of 5-HT1AR, activation and inhibition of 5-HT7R in the MDTN acutely enhanced and suppressed MK801-evoked thalamocortical glutamatergic transmission, respectively, without affecting basal L-glutamate release in the mPFC (Figure 1). Therefore, the excitatory effects of 5-HT7R in the MDTN on the thalamocortical glutamatergic pathway are possibly due to phasic activation [6,35].

Local administration of lurasidone into the MDTN acutely decreased MK801-evoked L-glutamate release in the mPFC (Figure 1). The binding affinity of lurasidone to 5-HT7R (Ki = 0.5 nM) is higher than that to 5-HT1AR (Ki = 6.8 nM) [37]. Considering the receptor binding profile of lurasidone, it is easily understood that the inhibitory effects of lurasidone are induced by a combination of 5-HT1AR partial agonism and 5-HT7R antagonism.

Under the condition of functional 5-HT1AR, acute local administration of escitalopram, which increases the extracellular serotonin level [6], into the MDTN did not affect MK801-evoked L-glutamate release in the mPFC (Figure 1), whereas under 5-HT1AR inhibition, escitalopram enhanced MK801-evoked L-glutamate release (Figure 2). Unlike that of escitalopram, under the condition of functional 5-HT1AR in the MDTN, local administration of vortioxetine inhibited MK801-evoked L-glutamate release in the mPFC (Figure 1), whereas under the condition of 5-HT1AR blockade, the inhibitory effects of vortioxetine continued to decrease (Figure 2). The binding affinities of serotonin to 5-HT1AR and 5-HT7R are in the order of nanomoles [57]. The binding affinity of vortioxetine to 5-HT3R (Ki = 1.1 nM) is higher than that to endogenous serotonin, but its affinity to 5-HT1AR (Ki = 230 nM) and 5-HT7R (Ki = 200 nM) is lower [39,40]. Furthermore, 5-HT3R is not expressed in the medial thalamic nuclei [66], whereas 5-HT7R is expressed predominantly in the MDTN [47,48]. Based on the serotonin receptor expression and vortioxetine binding profile, the inhibitory effects of vortioxetine on MK801-evoked L-glutamate release are predominantly generated by 5-HT7R antagonism, rather than 5-HT1AR or 5-HT3R. Although an in vitro study demonstrated the low affinity of vortioxetine to 5-HT7R compared to endogenous serotonin, vortioxetine could not inhibit 5-HT7R in rat brain. The present study also indicated the interesting pharmacologically opposite features of escitalopram and vortioxetine on the serotonergic regulation system in the thalamocortical glutamatergic pathway via modulation of 5-HT7R function.

### 3.2. Effects of Subchronic, Systemic Administration of Vortioxetine, Escitalopram, and Lurasidone on Thalamocortical Glutamatergic Transmission Associated with 5-HT7R

Similar to acute administration, subchronic systemic administrations of effective doses of vortioxetine and lurasidone inhibited MK801-evoked L-glutamate release, but that of escitalopram did not affect it (Figure 3). However, the subchronic administration of these three agents inhibited the stimulatory effects of local administration of 5-HT7R agonist (AS19) on MK801-evoked L-glutamate release in the mPFC (Figure 4). Therefore, subchronic administrations of effective doses of vortioxetine, escitalopram, and lurasidone suppressed the thalamocortical glutamatergic transmission associated with 5-HT7R due to downregulation of 5-HT7R in the MDTN (Figure 5 and Figure 6). Notably, subacute administrations of effective doses of vortioxetine and lurasidone for 3 days downregulated 5-HT7R in the MDTN (Figure 5). To the contrary, subacute administration of effective doses of escitalopram for 3 days did not affect the expression density of 5-HT7R in the MDTN, whereas escitalopram-induced downregulation of 5-HT7R required 7 days of subchronic administration of escitalopram (Figure 6). The discrepant duration for downregulation of 5-HT7R between the 5-HT7R inhibitors, vortioxetine and lurasidone, and escitalopram (an endogenous agonist enhancer via serotonin transporter inhibition), suggest different mechanisms of downregulation of 5-HT7R.

It has been demonstrated that chronic administration of fluoxetine (SSRI) for 21 days downregulates 5-HT7R in the rat hypothalamus [67]. Lurasidone has been considered to be a 5-HT7R antagonist, since lurasidone does not affect intracellular cyclic adenosine monophosphate (cAMP) accumulation, but it antagonised serotonin-induced cAMP accumulation in stable recombinant human 5-HT7R Chinese hamster ovary cells [37]. Vortioxetine also antagonised serotonin-induced cAMP accumulation in 5-HT7R-expressing HEK293 cells [68]. Similar to the results for lurasidone and vortioxetine, an adenylyl cyclase activity assay, using human 5-HT7R-expressing HEK293 cells, indicated that olanzapine and clozapine have 5-HT7R full inverse agonist-like features, but SB266970 is a 5-HT7R partial inverse agonist [69]. Interestingly, these three 5-HT7R inverse agonists downregulated 5-HT7R [69]. The downregulation of 5-HT7R induced by clozapine, olanzapine, and SB266970 and the present demonstrations, where subacute administration of effective doses of lurasidone and vortioxetine also downregulated 5-HT7R expression in the MDTN plasma membrane fraction, suggest that these agents are probably 5-HT7R inverse agonists but not antagonists. Furthermore, compared with the binding affinities of clozapine (Ki = 8.2 nM) and olanzapine (Ki = 365 nM) to 5-HT7R [70,71], the affinity of vortioxetine to rat 5-HT7R (Ki = 200 nM) is significantly lower than that to human 5-HT7R (Ki = 19 nM) [39,40], but the present results suggest that vortioxetine was able to suppress rat 5-HT7R function in vivo. It has been demonstrated that a heterodimer and homodimers composed of 5-HT1AR and 5-HT7R, together with monomers, coexist in the cells [72]. The heterodimer suppresses and enhances the stimulatory effects of 5-HT1AR on the G-protein-gated inwardly rectifying potassium channels and mitogen-activated protein kinases, respectively. Interestingly, the heterodimer enhances the internalisation of 5-HT1AR [72]. Considering that the highest affinity for complex formation was obtained for the 5-HT7R/5-HT7R homodimers, followed by the 5-HT7R/5-HT1AR heterodimers and 5-HT1AR/5-HT1AR homodimers, determination of the effects of vortioxetine and lurasidone on the functional interactions between the heterodimer, homodimers and monomers of 5-HT1AR and 5-HT7R could possibly clarify the complicated action of subchronic administration of these agents. The present study did not include the detailed mechanisms within its scope but indicated the rapid downregulation of 5-HT7R induced by inverse agonists (vortioxetine and lurasidone) compared with elevation of endogenous serotonin through serotonin transporter inhibition.

### 3.3. Clinical Implications of Vortioxetine, Escitalopram, and Lurasidone Associated with 5-HT7R

In cognitive function, it has been considered that the thalamus, including the MDTN, plays a role in the fundamental regulation of sensory integration [14,73]. The MDTN receives inputs from various cortical and subcortical regions associated with learning, memory, emotion, and perceptual integration [6,14,35,45,53,74,75,76]. The MDTN projects glutamatergic terminals to a number of cortical regions, such as the mPFC, insula, orbitofrontal cortex (OFC), and basal ganglia [6,14,35,45,53,54,55,77,78,79,80,81,82,83,84,85]. In spite of being feeble in rodents, the properties of thalamocortical glutamatergic transmission have been shown to translate from rodents to humans in terms of cognitive function [73,86].

It has been well established that the MDTN receives mainly inhibitory GABAergic inhibition from the RTN, which is activated by noradrenergic, serotonergic, and glutamatergic inputs via α1 adrenoceptor, 5-HT7R, and NMDA/glutamate receptors [6,35,45,53,54,55,78]. NMDA/glutamate receptor inhibitor, MK801, and ketamine/esketamine drastically enhanced thalamocortical glutamatergic transmission via intra-thalamic GABAergic disinhibition [6,35,54,55,56,82,87]. This forms the basis for fundamental scientific discussion on the pathophysiology of mood disorder and psychosis, since thalamocortical glutamatergic transmission is enhanced by ketamine/esketamine, which is effective in the treatment of antidepressant-resistant depression and schizophrenia-like psychosis inducers [14]. A number of clinical and preclinical studies displayed distinct hippocampal and thalamic non-dopaminergic mechanisms, playing important roles in ketamine-induced cognitive/memorial deficits [14]. Although there is no doubt that NMDA/glutamate receptor inhibition is the major mechanism of the antidepressive action of ketamine, ketamine-induced tonic GABAergic disinhibition in the MDTN probably elevates the threshold of sensory inputs, resulting in a disturbance to efficient sensory integration [6,14,35,53,55,56]. Therefore, conversion from tonic activation of thalamic activity induced by NMDA/glutamate receptor inhibition to phasic activation/inhibition possibly leads to the improvement of emotional perceptive/cognitive dysfunctions [14,78].

It is well known that the impairment of executive function is a critical antipsychotic-resistant cognitive domain of treatment-resistant schizophrenia [88], whereas lurasidone improves executive function in patients with atypical antipsychotic-resistant schizophrenia [43]. Interestingly, the approved dose of lurasidone (80 mg/day) improved executive functions in atypical antipsychotic-resistant schizophrenia better than higher doses; however, the effect of lurasidone on executive function was independent of improvements in the positive and negative syndrome scales [89]. Therefore, the improvement of executive functions (atypical antipsychotic-resistant cognitive domains) in treatment-resistant schizophrenia by a relatively low dose of lurasidone suggests that 5-HT7R antagonism plays an important role in the cognition-promoting effects of lurasidone, rather than 5-HT2A antagonism or 5-HT1AR partial agonism.

SSRIs, currently the first-line pharmacologic treatment for depression, fails to improve emotional perception/cognition dysfunctions in individuals with major depression, such as sustained, selective, and divided attention [90,91,92]. Furthermore, a systematic review of healthy individuals reported that SSRIs worsened divided and sustained attention [93]. Contrarily to SSRIs, vortioxetine improved sustained and selective attention during depressive moods in individuals with major depression [94,95]. Considering these preclinical and clinical findings, the present results suggest the possibility that direct inhibition of thalamic 5-HT7R contributes to the mechanisms of improvement of deficits in emotional perception and attention, but downregulation of 5-HT7R cannot provide these. To clarify our hypothesis, the impact of the interaction between thalamocortical glutamatergic transmission and intra-thalamic serotonergic transmission associated with 5-HT7R should be determined in further studies.

## 4. Materials and Methods

### 4.1. Chemical Agents

Lurasidone, the 5-HT7R antagonist SB269970, and the non-competitive NMDA-R antagonist MK801 were obtained from Fujifilm-Wako (Osaka, Japan). Vortioxetine, escitalopram, and the 5-HT7R agonist, AS19, were purchased from Cosmo-Bio (Tokyo, Japan). All compounds were prepared on the day of experiments. All drugs were perfused in modified Ringer’s solution (MRS): 145 mM Na^+^, 2.7 mM K^+^, 1.2 mM Ca^2+^, 1.0 mM Mg^2+^, and 154.4 mM Cl^−^, adjusted to pH 7.4 using 2 mM phosphate buffer and 1.1 mM Tris buffer.

Escitalopram, MK801, and SB269970 were dissolved in MRS directly. Vortioxetine and AS19 were initially dissolved in dimethyl sulfoxide at 25 mM. Lurasidone was initially dissolved in dimethyl sulfoxide at 1 mg/mL. The final dimethyl sulfoxide concentration was lower than 0.1% (*v*/*v*).

Previous studies have reported that the minimum effective doses of systemic administrations of vortioxetine and escitalopram on the extracellular serotonin level are 2.5 mg/kg [68] and 5 mg/kg/day [62], respectively. The effective dose of subchronic administration of lurasidone is 3 mg/kg/day [6]. According to these previous reports, in the present study, to study the subchronic effects of lurasidone, vortioxetine, and escitalopram, rats were subcutaneously administered lurasidone (3 mg/kg/day), vortioxetine (2.5 mg/kg/day), or escitalopram (5 mg/kg/day) for 3 or 7 days using an osmotic pump (2ML_1, Alzet, Cupertino, CA, USA). The present study used concentrations of AS19 (5 μM), SB269970 (10 μM), and MK801 (10 μM) according to previous studies [6,35,45,54,96]

### 4.2. Microdialysis System

All animal care and experimental procedures were performed in compliance with the ethical guidelines established by the Institutional Animal Care and Use Committee at Mie University (No. 29–30). All studies involving animals are reported in accordance with the relevant ARRIVE guidelines [97] and European Union Council regulations (2010/63/EU). A total of 84 rats were used in experiments.

Male Sprague–Dawley rats (approximately 250 g, 7−8 weeks old, SLC, Shizuoka, Japan) were maintained in a controlled environment (22 °C ± 1 °C) with a 12-h light/12-h dark cycle. Especially, to study the subchronic effects, male Sprague–Dawley rats (approximately 200 g, 7 weeks old, SLC) were administered lurasidone (3 mg/kg/day), vortioxetine (2.5 mg/kg/day), or escitalopram (5 mg/kg/day) for 3 or 7 days using a subcutaneous osmotic pump (2ML_1, Alzet). The nominal pumping rate and duration of the 2ML_1 osmotic pump were 10 μL/h and 7 days.

All rats were weighed before the study. Rats were anesthetized with 1.8% isoflurane and were then placed in a stereotaxic frame for implantation of microdialysis probes. Concentric direct insertion-type dialysis probes (0.22 mm diameter; Eicom, Kyoto, Japan) were implanted in the medial prefrontal cortex (mPFC: 3 mm exposed membrane; A = +3.2 mm, L = +0.8 mm, V = −5.2 mm, relative to the bregma), the mediodorsal thalamic nucleus (MDTN: 2 mm exposed membrane; A = −3.0 mm, L = +0.9 mm, V = −6.2 mm, relative to the bregma at a lateral angle of 30°), and the reticular thalamic nucleus (RTN: 2 mm exposed membrane: A = −1.4 mm, L = +1.2 mm, V = −7.2 mm, relative to bregma) [98]. During recovery and experimentation, rats were housed individually in cages and were provided food and water ad libitum. Perfusion experiments were initiated at 18 h after recovery from isoflurane anaesthesia [99,100,101]. During experiments, rats were placed in an in vivo dialysis system for freely moving animals (Eicom), equipped with a two-channel swivel (TCS2-23; ALS, Tokyo, Japan). The perfusion rate was set at 2 μL/min in all experiments using MRS [101], and dialysates were collected over 20 min sampling epochs. Extracellular l-glutamate levels were measured at 8 h after the start of the perfusions. After baseline recording, the perfusion medium was switched to MRS containing MK801 (10 μM), lurasidone (1 μM), vortioxetine (20 μM), escitalopram (10 μM), AS19 (5 μM), or SB269970 (10 μM), as indicated. Dialysate samples were then injected into the UHPLC apparatus. All samples were taken from freely moving animals.

### 4.3. Determination of Extracellular L-Glutamate Level

The concentration of L-glutamate in the perfusate (μM) was determined using UHPLC (xLC3185PU; Jasco, Tokyo, Japan) with fluorescence resonance energy transfer detection (xLC3120FP; Jasco) after dual derivatization with isobutyryl-l-cysteine and *o*-phthalaldehyde. Derivative reagent solutions were prepared by dissolving isobutyryl-l-cysteine (2 mg) or *o*-phthalaldehyde (1 mg) in 0.1 mL aliquots of ethanol, followed by addition of 0.9 mL of sodium borate buffer (0.2 M, pH 9.0) [102]. Automated precolumn derivation was conducted by mixing 5 μL sample, standard, or blank solutions with 5 μL of derivative reagent solution in reaction vials for 5 min before injection. Derivative samples (5 μL) were injected using an autosampler (xLC3059AS; Jasco). The analytical column (YMC Triart C18, particle 1.8 μm, 50 × 2.1 mm; YMC, Kyoto, Japan) was maintained at 45 °C. The flow rate was set at 500 μL/min, and elution was performed using a linear gradient of mobile phases A (0.05 M acetate buffer, pH 5.0) and B (0.05 M acetate buffer containing 60% acetonitrile, pH 3.5) over 10 min [103]. Excitation and emission wavelengths of the fluorescence detector were set at 280 and 455 nm, respectively.

Where possible, we randomized and blinded the sample data. In particular, for determinations of the extracellular transmitter levels, the sample order was dictated by the autosampler according to a random number table.

### 4.4. Capillary Immunoblotting Analysis

Total plasma membrane proteins of the thalamus were extracted using the Minute Plasma Membrane Protein Isolation Kit (Invent Biotechnologies, Plymouth, MN). The capillary immunoblotting analyses were performed using Wes (ProteinSimple, Santa Clara, CA, USA) according to the ProteinSimple user manual [77,78,79,80,81,104]. The lysates of the plasma membrane fraction were mixed with a master mix (ProteinSimple) to a final concentration of 1 × sample buffer, 1 × fluorescent molecular weight marker, and 40 mM dithiothreitol and then heated at 95 °C for 5 min. The samples, blocking reagents, primary antibodies, HRP-conjugated secondary antibodies, chemiluminescent substrate (SuperSignal West Femto: Thermo Fisher Scientific, Waltham, MA, USA), and separation and stacking matrices were also dispensed to the designated wells in a 25-well plate. After plate loading, the separation electrophoresis and immunodetection steps took place in the capillary system and were fully automated. A capillary immunoblotting analysis was carried out at room temperature, and the instrument’s default settings were used. Capillaries were first filled with a separation matrix followed by a stacking matrix, with about 40 nL of the sample used for loading. During electrophoresis, the proteins were separated by molecular weight through the stacking and separation matrices at 250 volts for 40–50 min and then immobilized on the capillary wall using proprietary photo-activated capture chemistry. The matrices were then washed out. The capillaries were next incubated with a blocking reagent for 15 min, and the target proteins were immunoprobed with primary antibodies followed by HRP-conjugated secondary antibodies (Anti-Rabbit IgG HRP, A00098, 10 μg/mL, GenScript, Piscataway, NJ, USA). The antibodies of GAPDH (NB300-322, 1:100, Novus Biologicals, Littleton, CO, USA) and 5-HT7R (NB100-56352, Novus Biologicals) were diluted in an antibody diluent (ProteinSimple).

### 4.5. Data Analysis

Where possible, we randomized and blinded the sample data. To determine the extracellular transmitter levels, the sample order was set on the autosampler according to a random number table. Drug doses and sample sizes were selected according to previous studies [35,55,82]. All experiments in this study were designed with equally sized animal groups (*n* = 6) [35,82,87,105] and all values were expressed as the means ± standard deviations (SD). Differences were considered significant when *p* < 0.05 (two-tailed).

Regional transmitter concentrations were analysed using Mauchly’s sphericity test followed by multivariate analysis of variance (MANOVA) using BellCurve for Excel ver. 3.20 (Social Survey Research Information Co., Ltd., Tokyo, Japan). The data were composed of the average of the pre-treatment period (1 point) and each time point during target agent administration (9 points). When the data did not violate the assumption of sphericity (*p* > 0.05), the F-value of the MANOVA was analysed using sphericity-assumed degrees of freedom. When the assumption of sphericity was violated (*p* < 0.05), F-values were analysed using Chi–Muller’s corrected degrees of freedom by BellCurve for Excel. When the F-values for the drug factors were significant in MANOVA, the data were finally analysed using Tukey’s post hoc test with BellCurve for Excel. Transmitter levels were expressed as the area under the curve between 20 and 180 min (AUC20–180 min) after target agent administration. All statistical analyses complied with the recommendations for experimental design and analysis in pharmacology [106].

The protein expression of 5-HT7R in the thalamic plasma membrane fraction was analysed by a one-way ANOVA with Tukey’s multiple comparison using BellCurve for Excel.

## 5. Conclusions

The present study demonstrated that thalamocortical glutamatergic transmission was regulated by inhibitory 5-HT1AR and excitatory 5-HT7R, but regulation of 5-HT7R, rather than 5-HT1AR, was predominant in the MDTN. Both vortioxetine and lurasidone acutely inhibited thalamocortical glutamatergic transmission due to direct inhibition of 5-HT7R rather than 5-HT1AR activation, whereas escitalopram acutely activated both inhibitory 5-HT1AR and excitatory 5-HT7R due to an increase in the extracellular serotonin level through serotonin transporter inhibition, resulting in offset effects on the thalamocortical glutamatergic transmission. Similar to acute administration, subchronic vortioxetine and lurasidone attenuated thalamocortical glutamatergic transmission, but escitalopram did not affect it, whereas subchronic administration of these three agents suppressed excitatory 5-HT7R via 5-HT7R downregulation in the MDTN. 5-HT7R was downregulated by subchronic administration of vortioxetine, escitalopram, and lurasidone, but the downregulation of 5-HT7R induced by the 5-HT7R inhibitors (inverse agonists) vortioxetine and lurasidone was rapid compared to that by escitalopram. These discrepancies between the 5-HT7R inverse agonists (vortioxetine and lurasidone) and the SSRI (escitalopram) on thalamocortical glutamatergic transmission associated with 5-HT7R inhibition might, at least partially, contribute to their effectiveness on emotional/mood perception and risk of initial adverse reactions, such as jitteriness/anxiety syndrome.

## Data Availability

The data presented in this study are available on request from the corresponding author. The data are not publicly available due to equipment-dependent data.
